# Adherence to prescribing restrictions for HER2-positive metastatic breast cancer in Australia: A national population-based observational study (2001-2016)

**DOI:** 10.1371/journal.pone.0198152

**Published:** 2018-07-26

**Authors:** Benjamin Daniels, Federico Girosi, Hanna Tervonen, Belinda E. Kiely, Sarah J. Lord, Nehmat Houssami, Sallie-Anne Pearson

**Affiliations:** 1 Medicines Policy Research Unit, Centre for Big Data Research in Health, UNSW, Sydney, Australia; 2 Translational Health Research Institute, Western Sydney University, Penrith, New South Wales, Australia; 3 Capital Markets CRC, Sydney, New South Wales, Australia; 4 NHMRC Clinical Trials Centre, University of Sydney, Sydney, Australia; 5 School of Medicine, University of Notre Dame Australia, NHMRC Clinical Trials Centre, University of Sydney, Sydney, Australia; 6 Sydney School of Public Health, Sydney Medical School, University of Sydney, Sydney, Australia; University of South Alabama Mitchell Cancer Institute, UNITED STATES

## Abstract

**Background:**

Targeted cancer therapy is often complex, involving multiple agents and chemotherapeutic partners. In Australia, prescribing restrictions are put in place to reflect existing evidence of cost-effectiveness of these medicines. As therapeutic options continue to expand, these restrictions may not be perceived to align with best practice and it is not known if their use in the real-world clinic adheres to these restrictions. We examined the treatment of women receiving trastuzumab for HER2-positive metastatic breast cancer (HER2+MBC) to determine the extent to which treatment adhered to national prescribing restrictions.

**Patients and methods:**

Our population-based, retrospective cohort study used dispensing records for every Australian woman initiating publicly-subsidised trastuzumab for HER2+MBC between 2001–2013, followed through 2016. We used group-based trajectory models (GBTMs) to cluster patients, first on their patterns of trastuzumab exposure, and then on their patterns of lapatinib and chemotherapy exposure. We described the characteristics of patients within each cluster, and examined their treatments and combinations of treatments to determine restriction adherence.

**Results:**

Of 5,052 patients initiating trastuzumab, 1,795 (36%) received at least one non-adherent HER2-targeted treatment. The most common non-adherent treatments were trastuzumab combinations involving vinorelbine (24% of non-adherent treatments); capecitabine (24%); and anthracyclines (10%). Non-adherent lapatinib use was observed in 4% of patients. GBTM identified three trastuzumab exposure clusters, each containing three further sub-clusters. The largest proportions of non-adherent treatments were in sub-clusters with longer trastuzumab exposure and more non-taxane chemotherapy. Patients in these sub-clusters were younger than those in sub-clusters with less non-adherent treatment.

**Conclusions:**

Our study highlights that, even during the relatively simpler treatment era of our study period, a substantial amount of treatment did not adhere to prescribing restrictions. As more trials are conducted exploring pertuzumab and T-DM1 in combination with different chemotherapies and other HER2-targeted therapies, the regulation and funding of HER2-targeted treatment will become more challenging.

## Introduction

The discovery and use of trastuzumab over the past two decades has revolutionised the treatment and survival of patients with HER2-positive metastatic breast cancer (HER2+MBC).[1] More recently, clinical trials of lapatinib, pertuzumab, and trastuzumab emtansine (T-DM1) have demonstrated further survival gains.[2–4] Currently, clinicians have “an abundance of riches”[5] in terms of HER2-targeted, antineoplastic, and hormonal treatment options for patients with HER2+ metastatic breast cancer. Despite this, little is known about how these treatments are sequenced and combined in the “real-world” setting, outside of clinical trials.

In Australia, HER2-targeted therapies are publicly-subsidised for HER2+MBC with specific restrictions governing their use.[6] These restrictions are based on evidence from the pivotal clinical trials of these medicines,[2–4, 7, 8] and are designed to ensure that the medicines are used in ways deemed cost-effective. Our own research suggests that between 2001 and 2015 there was substantial heterogeneity in the use of trastuzumab for HER2+MBC in terms of duration of therapy and chemotherapeutic partners, leading us to believe prescribing restrictions were often not adhered to.[9] The evidence base for effective treatments is constantly evolving, and prescribing restrictions may often lag behind the most recent evidence, but the extent of variation in HER2+MBC treatment and adherence to prescribing restrictions are currently unknown.

Therefore, in the present study we describe the treatment pathways for patients starting trastuzumab treatment for HER2+MBC in Australia with the aim of determining how often HER2-targeted treatment adhered to prescribing restrictions. We clustered patients based on trajectories of trastuzumab, lapatinib, and chemotherapy use; described the characteristics of each cluster; and compared the observed treatments within each cluster to national prescribing restrictions to determine how closely treatment adhered to these restrictions.

## Patients & methods

### Setting and data

The Australian healthcare setting and the datasets used in this study have been described in our research protocol.[6] Briefly, Australia maintains a publicly funded, universal healthcare system entitling all citizens and permanent residents to subsidised medicines through the Pharmaceutical Benefits Scheme (PBS). The Herceptin Program, a separate funding program sitting outside of the PBS, provided subsidised access to trastuzumab for HER2+MBC from December 2001 until July 2015, when the program was closed and trastuzumab for HER2+MBC was listed for subsidy on the PBS.[10]

The Department of Human Services (DHS)—administering body for the Herceptin Program and the PBS–supplied de-identified patient-level data for every woman accessing publicly-subsidised trastuzumab, lapatinib, pertuzumab, and T-DM1 for MBC in Australia between 3 December 2001 and 30 June 2016. The datasets provided by DHS were: Herceptin Program enrolment data including year of birth and month/year of death; dispensed trastuzumab records including dates and quantity supplied; and PBS dispensing records detailing other medicines dispensed. The DHS also provided the dispensing records for all patients in Australia who accessed publicly-subsidised trastuzumab for early breast cancer (EBC) from 1 October 2006 to 30 June 2016. We determined previous treatment with trastuzumab for EBC through data linkage of Herceptin Program records with the dispensing records of patients who received trastuzumab for EBC.

The full period of time observed across the datasets is 1 January 2001 to 30 June 2016.

### Study design and participants

Our population-based, retrospective cohort study includes every Australian woman initiating trastuzumab for MBC subsidised through the *Herceptin Program* between 3 December 2001 and 30 June 2013, followed until death or 30 June 2016. We chose this study period to allow for a minimum of three years potential observation time for each patient. In Australia, once a medicine is subsidised through the PBS or the *Herceptin Program*, the government bears the cost of the medicine. Private insurance will not provide reimbursement for medicines already subsidised on these programs so that it is unlikely that patients would access these medicines through other avenues and it would not be necessary for them to pay for the entire cost-of the medicine out of their own pocket. As such, our study population likely captures all patients in Australia treated with HER2-targeted therapies for MBC during the study period except for those accessing these medicines as a part of a clinical trial.

### Outcomes and statistical analysis

#### Clustering patients based on patterns of trastuzumab, lapatinib, and chemotherapy exposure

**Fig 1:**
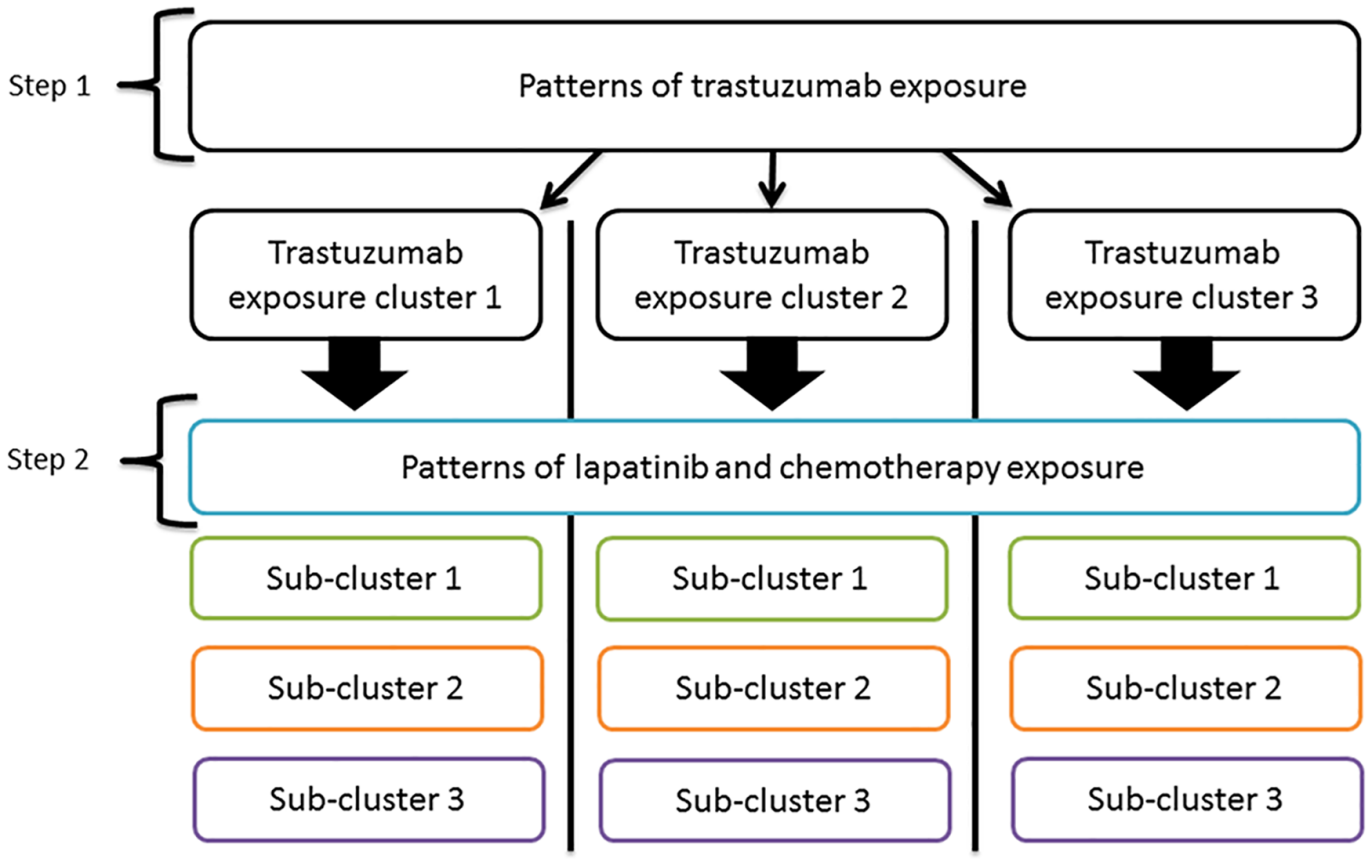
Schematic detailing the hierarchical framework by which patients were clustered. Step 1: Patients were clustered according to their patterns of trastuzumab exposure; Step 2: Within the resulting trastuzumab exposure clusters, patients were then further clustered according to their patterns of lapatinib and chemotherapy exposure. The end result was 9 distinct sub-clusters.

In preliminary analyses, we found the number of treatments and treatment combinations unmanageable and difficult to interpret. Therefore, in order to provide a framework for our subsequent analyses, we clustered patients based on monthly patterns of trastuzumab, lapatinib, and chemotherapy dispensings for the first three years (36 months) after starting trastuzumab for MBC. We performed the clustering using a hierarchical approach as illustrated in Fig 1.

Because all patients in our cohort received trastuzumab and our definition of adherence largely depended on trastuzumab exposure, we first clustered patients based on trajectories of trastuzumab exposure; then, within the resulting trastuzumab exposure clusters, we further clustered patients based on their trajectories of other antineoplastics use, including lapatinib (ATC code: L01XE07), taxanes (paclitaxel [L01CD01], docetaxel [L01CD02]), capecitabine (L01BC06), platinum agents (cisplatin [L01XA01], carboplatin [L01XA02]), gemcitabine (L01BC05), and vinorelbine (L01CA04). Pertuzumab and T-DM1 were excluded from the clustering analysis because they were not publicly-subsidised in Australia until July 2015 and few patients in our cohort received these medicines during their first three years of treatment (7% received pertuzumab; 8% received T-DM1).

We used logistic group-based trajectory modeling (GBTM) and group-based multi-trajectory modeling (GBMTM) to cluster patients.[11, 12] GBTMs/GBMTMs are unsupervised models that identify latent clusters of patients following similar trajectories across longitudinal outcomes. We created 36 binary variables (one for each month) for each treatment where “1” indicated a dispensing of that treatment in the given month; “0” indicated no dispensing in the given month; missing values indicated that a patient had died. We explored 3–7 latent clusters for each level of the hierarchy, theorising that less than three would be too few to capture potentially meaningful variation and more than seven would be difficult to meaningfully interpret. All models were adjusted for patient age at initiation, year of initiation, and prior exposure to trastuzumab for adjuvant therapy (yes/no). To account for patient drop-out due to death we allowed attrition rates to be modelled along with the trajectories.[13] We determined the optimal number of clusters by evaluating model fit using the Bayesian information criterion for all models.

#### Treatment courses and the treatment pathway

We identified patient clusters based on treatment exposure patterns during the first three years of therapy and then created treatment courses, within each cluster, based on the entirety of the observed dispensing data for each patient (observed until 30 June 2016). We defined a treatment course for each medicine as the period from first dispensing date of that medicine until the last dispensing date, plus 30 days or the number of days to death, whichever was sooner. We considered a period of >90 days between dispensings as a break in treatment and a dispensing following a break of >90 days as beginning a new course of therapy.[14] For each patient, we considered treatment courses of different medicines that overlapped in time for ≥30 days as distinct combination therapies. We used these treatment courses to identify instances of adherent and non-adherent treatment for each patient.

#### Summary of prescribing restrictions

Between 2001–2015, the use of publicly-funded trastuzumab was restricted to monotherapy or combination therapy with a taxane. Trastuzumab had to be ceased at the time of disease progression. When lapatinib was publicly-subsidised in 2008, its use was restricted to combination therapy with capecitabine after prior trastuzumab. From 2015, the restrictions were changed allowing trastuzumab to be combined with any chemotherapy (except nanoparticle aluminium-bound [nab] paclitaxel and eribulin) and continued beyond progression. From July 2015, pertuzumab was funded for use as first-line therapy in combination with trastuzumab and a taxane; and T-DM1 was funded as monotherapy after treatment with pertuzumab.[6]

Based on these restrictions, we defined the following treatment as non-adherent:

1. Trastuzumab used in combination with non-taxane chemotherapy prior to 1 July 2015.
2. Trastuzumab used in combination with lapatinib.
3. Lapatinib initiated and continued for at least 30 days as monotherapy or used in combination with any chemotherapy other than capecitabine.
4. Pertuzumab used without concurrent trastuzumab and a taxane
5. T-DM1 used in combination with any chemotherapy or HER2-targeted therapies

Herceptin Program and PBS data do not contain specific information identifying disease progression and proxies for progression using these datasets have been shown to be unreliable.[15] It is recommended practice to change a chemotherapeutic agent at the time of disease progression, [16] and during the study period it was also common practice to continue trastuzumab treatment beyond disease progression but change chemotherapeutic agents.[17] The use of chemotherapy agents with trastuzumab following treatment with a taxane in our cohort likely represents trastuzumab continued beyond progression, however, we did not attempt to identify specific instances of the non-adherent practice of continuing trastuzumab use beyond disease progression. All chemotherapy and chemotherapy combinations not including HER2-targeted therapies were classified as adherent for the purpose of this analysis.

#### Statistical analyses

For each patient cluster we used descriptive statistics to summarise: age, fact of death, the number of treatments patients received, and the number of non-adherent treatments patients received and the number of patients with at least one non-adherent treatment. We estimated OS from the date of first trastuzumab dispensing for HER2+MBC until month of death (set at the last day of the month) or censor (30 June 2016) using Kaplan-Meier methods. All analyses were performed in SAS version 9.4 (SAS Institute, Cary, NC) with figures generated using R v3.3.3,[18] ggplot2,[19] and sunburstR.[20]

### Ethics and data access approvals

Our study was approved by the NSW Population and Health Services Research Ethics Committee (Approval Number: 2010/02/213) and data access was granted by the Australian Department of Human Services (DHS) External Request Evaluation Committee (Approval Numbers: MI1474, MI1475, MI1477, MI5858). Individual consent for the release of these data has been waived according to the Australian Privacy Act of 1988[6]. Direct access to the data and analytical files to other individuals or authorities is not permitted without the express permission of the approving human research ethics committees and data custodians.

## Results

There were 5,052 patients who initiated trastuzumab for MBC between 3 December 2001 and 30 June 2013. Overall, 1,795 patients (36%) received non-adherent HER2-targeted treatment at some point during therapy (Table 1). The most frequent non-adherent treatments were trastuzumab plus: vinorelbine (24%); capecitabine (24%); anthracycline (10%); and taxane with platinum (TCH; 9%). A total of 193 patients (4%) received non-adherent lapatinib treatment: 165 patients initiated lapatinib as monotherapy while 28 patients received lapatinib in combination with chemotherapy other than capecitabine. Non-adherent concomitant therapy with trastuzumab and lapatinib was observed in 37 patients (<1%) who had dispensings of lapatinib while continuing trastuzumab treatment.

**Table 1:** Patient characteristics and non-adherent treatments for the entire cohort and according to trastuzumab exposure clusters.

	All patients	Trastuzumab exposure Cluster 1	Trastuzumab exposure Cluster 2	Trastuzumab exposure Cluster 3
Patients, n	5,052	1,346	1,500	2,206
Baseline measures				
Age at first trastuzumab for MBC dispensing, median (IQR)	56 (48–65)	57 (49–67)	55 (47–64)	56 (48–66)
Year of trastuzumab for MBC initiation, median (IQR)	2007 (2004–2010)	2006 (2004–2010)	2007 (2005–2010)	2008 (2005–2010)
Previously treated with trastuzumab for EBC, n (%)	531 (11)	180 (13)	139 (9)	212 (10)
Post-trastuzumab initiation measures				
Number of treatment courses per patient, median (IQR)	2 (1–4)	2 (1–3)	2 (1–4)	3 (1–5)
Non-adherent treatment (any):				
Treatment courses, n (% of administered courses)	2,828 (17)	356 (10)	875 (16)	1,597 (20)
Patients, n (% of patients in each cluster)	1,795 (36)	292 (22)	604 (40)	899 (41)
Non-adherent trastuzumab treatment:				
Treatment courses, n (%)	2,594 (15)	302 (9)	777 (14)	1,515 (19)
Patients, n (%)	1,660 (33)	255 (19)	545 (36)	860 (39)
Non-adherent lapatinib treatment:				
Treatment courses, n (%)	193 (1)	50 (1)	82 (2)	61 (1)
Patients, n (%)	193 (4)	50 (4)	82 (5)	61 (3)

**Fig 2:**
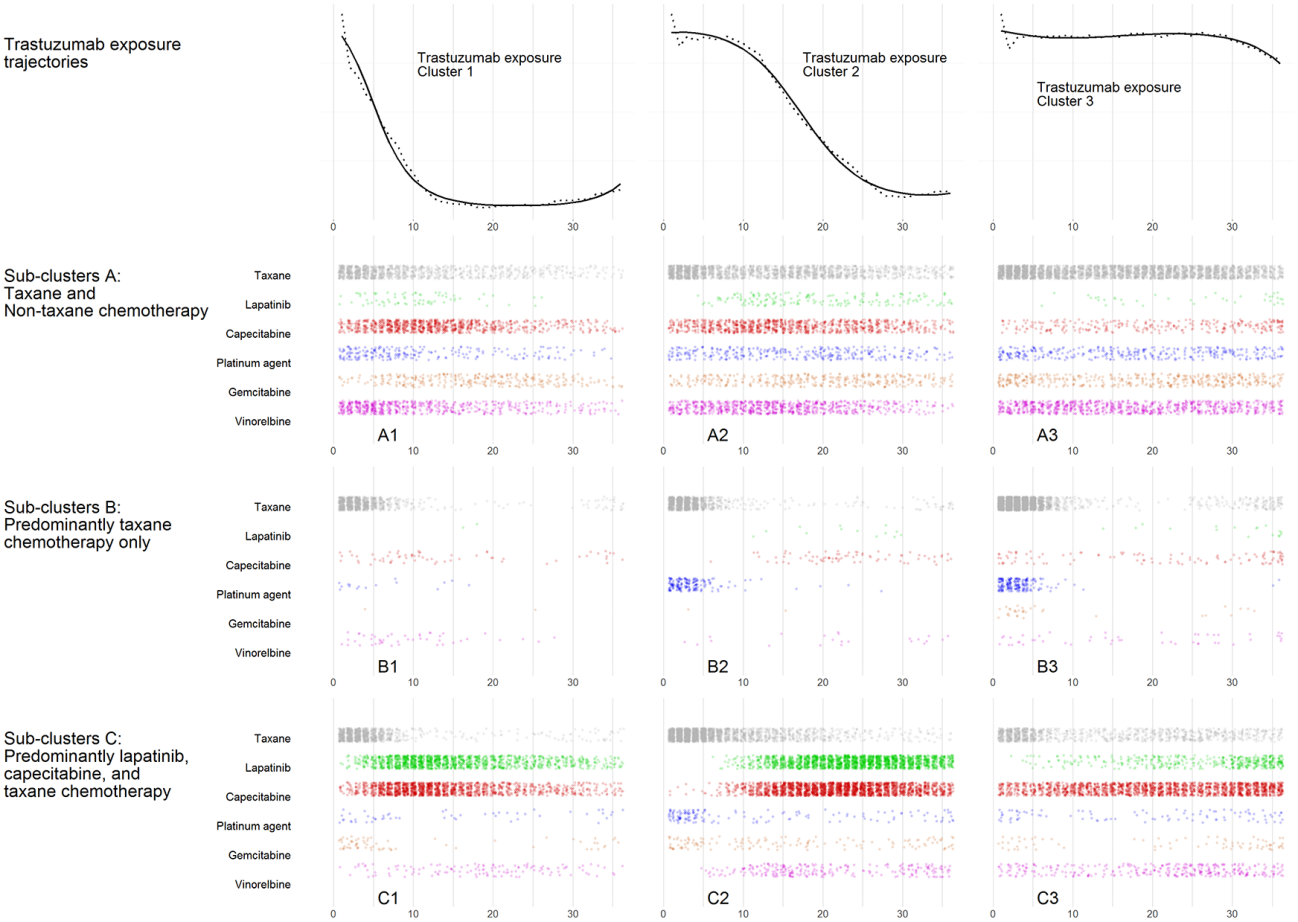
Antineoplastic use by sub-cluster. Trastuzumab exposure trajectories (top row) showing the predicted (solid line) exposure trajectory (proportion of patients in each month with a trastuzumab dispensing) and observed exposure trajectory (dotted line) during the 36 months from initiation; and dot plots (rows 2–4) showing the number of patients in each month with a dispensing of additional antineoplastic therapies, within each trastuzumab exposure trajectory cluster.

GBTM identified three distinct trastuzumab exposure trajectories (top row, Fig 2) broadly clustering patients as those who:

1. Discontinued or died within 12 months of initiation;
2. Discontinued or died between 12 to 24 months from initiation (some may have had breaks in therapy); and
3. Continued until death or the entire 36-month clustering period.

Within each trastuzumab exposure cluster, GBMTM identified three further sub-clusters based on patterns of antineoplastic exposure (Fig 2, rows 2–4). These sub-clusters are generally described as patients who received:

1. Taxane and non-taxane chemotherapy
2. Predominantly taxane chemotherapy only; and
3. Predominantly lapatinib, capecitabine, and taxane chemotherapy

We present the characteristics of each cluster and sub-clusters in Tables 1 and 2. Patients in Sub-clusters B1 –B3 were older than those in Sub-clusters A1 –A3 and C1 –C3, while patients in Sub-clusters C1 –C3 initiated trastuzumab later than those in Sub-clusters A1 –A3 and B1 –B3 (Table 2). Patients in sub-clusters A1 –A3 and C1 –C3 had the highest proportions of non-adherent treatment, ranging from 16% to 31% of administered treatment, with between 33%–81% of patients in these sub-clusters receiving at least one non-adherent treatment (Table 2).

**Table 2:** Patient characteristics and non-adherent treatment by antineoplastic exposure sub-clusters within trastuzumab exposure clusters.

	Trastuzumab exposure clusters		
	Cluster 1 (n = 1,346)	Cluster 2 (n = 1,500)	Cluster 3 (n = 2,206)
Sub-clusters A: Taxane and non-taxane chemotherapy	A1	A2	A3
n	382	473	487
Age at first trastuzumab for MBC dispensing, median (IQR)	55 (48–63)	53 (45–60)	54 (46–62)
Year of trastuzumab for MBC initiation, median (IQR)	2005 (2003–2007)	2006 (2003–2009)	2007 (2004–2010)
Previously treated with trastuzumab for EBC, n (%)	31 (8)	49 (10)	57 (12)
% died	99%	97%	84%
Total number of treatment courses	1,310	1,967	2,457
Number of treatment courses per patient, median (IQR)	2 (1–3)	3 (2–4)	3 (2–6)
Number of distinct chemotherapy agents dispensed per patient, median (IQR)	2 (1–3)	2 (1–3)	2 (1–3)
Non-adherent treatment:			
Non-adherent treatment courses, n (% of treatments within sub-cluster)	203 (16)	445 (23)	624 (25)
Non-adherent initiation treatment course, n (% of initiation treatment courses within sub-cluster)	99 (26)	134 (28)	127 (26)
Later non-adherent treatment courses, n (% of treatment courses within sub-cluster)	108 (8)	311 (16)	497 (20)
Patients with at least one non-adherent treatment course, n (% of sub-cluster)	161 (42)	279 (59)	316 (65)
Proportion of survival time spent on non-adherent treatment courses, median (IQR)	33% (18%–52%)	27% (13%–50%)	25% (14%–48%)
Median time on trastuzumab, months (IQR)	5.8 (3.5–8.8)	14.1 (8.0–19.4)	27.1 (10.5–43.0)
Median overall survival, months (IQR)	15.1 (10.1–24.6)	26.7 (18.3–35.2)	41.2 (19.1–66.0)
Sub-clusters B: Predominantly taxane chemotherapy only	B1	B2	B3
n	658	585	1,404
Age at first trastuzumab for MBC dispensing, median (IQR)	61 (51–74)	58 (49–66)	58 (48–68)
Year of trastuzumab for MBC initiation, median (IQR)	2005.5 (2003–2009)	2007 (2005–2010)	2008 (2005–2011)
Previously treated with trastuzumab for EBC, n (%)	55 (8)	28 (5)	122 (9)
% died	88%	58%	60%
Total number of treatment courses	1,123	1,402	3,771
Number of treatment courses per patient, median (IQR)	1 (1–2)	2 (1–3)	2 (1–4)
Number of distinct chemotherapy agents dispensed per patient, median (IQR)	1 (0–1)	1 (0–1)	1 (0–1)
Non-adherent treatment:			
Non-adherent treatment courses, n (% of treatments within sub-cluster)	33 (3)	138 (10)	472 (13)
Non-adherent initiation treatment course, n (% of initiation treatment courses within sub-cluster)	21 (3)	83 (14)	155 (11)
Later non-adherent treatment courses, n (% of treatment courses within sub-cluster)	12 (1)	55 (4)	317 (8)
Patients with at least one non-adherent treatment course, n (% of sub-cluster)	30 (5)	111 (19)	329 (23)
Proportion of survival time spent on non-adherent treatment courses, median (IQR)	8% (3%–15%)	7% (4%–15%)	9% (4%–19%)
Median time on trastuzumab, months (IQR)	4.3 (1.9–7.4)	14.9 (12.5–18.5)	33.1 (7.2–63.5)
Median overall survival, months (IQR)	11.7 (6.6–23.4)	42.6 (21.8 –NR*)	63.3 (11.2–139.3)
Sub-clusters C: Predominantly lapatinib, capecitabine, and taxane chemotherapy	C1	C2	C3
n	306	442	315
Age at first trastuzumab for MBC dispensing, median (IQR)	54 (47–62)	54 (47–61)	55 (46–64)
Year of trastuzumab for MBC initiation, median (IQR)	2009 (2007–2011)	2008 (2006–2011)	2008 (2005–2010)
Previously treated with trastuzumab for EBC, n (%)	94 (31)	62 (14)	33 (10)
% died	94%	93%	85%
Total number of treatment courses	1,080	2,054	1,640
Number of treatment courses per patient, median (IQR)	2 (1–4)	3 (2–4)	3 (2–5)
Number of distinct chemotherapy agents dispensed per patient, median (IQR)	2 (2–3)	2 (2–3)	2 (2–3)
Non-adherent treatment:			
Non-adherent treatment courses, n (% of treatments within sub-cluster)	120 (11)	292 (14)	501 (31)
Non-adherent initiation treatment course, n (% of initiation treatment courses within sub-cluster)	32 (11)	62 (14)	113 (36)
Later non-adherent treatment courses, n (% of treatment courses within sub-cluster)	88 (8)	230 (11)	388 (24)
Patients with at least one non-adherent treatment course, n (% of sub-cluster)	101 (33)	214 (48)	254 (81)
Proportion of survival time spent on non-adherent treatment courses, median (IQR)	17% (12%–27%)	16% (9%–26%)	33% (17%–54%)
Median time on trastuzumab, months (IQR)	6.7 (4.3–8.6)	15.4 (12.3–19.8)	28.7 (12.7–35.7)
Median overall survival, months (IQR)	15.7 (10.6–25.5)	28.3 (20.6–42.5)	42.8 (30.0–58.9)

*NR = not reached

**Fig 3:**
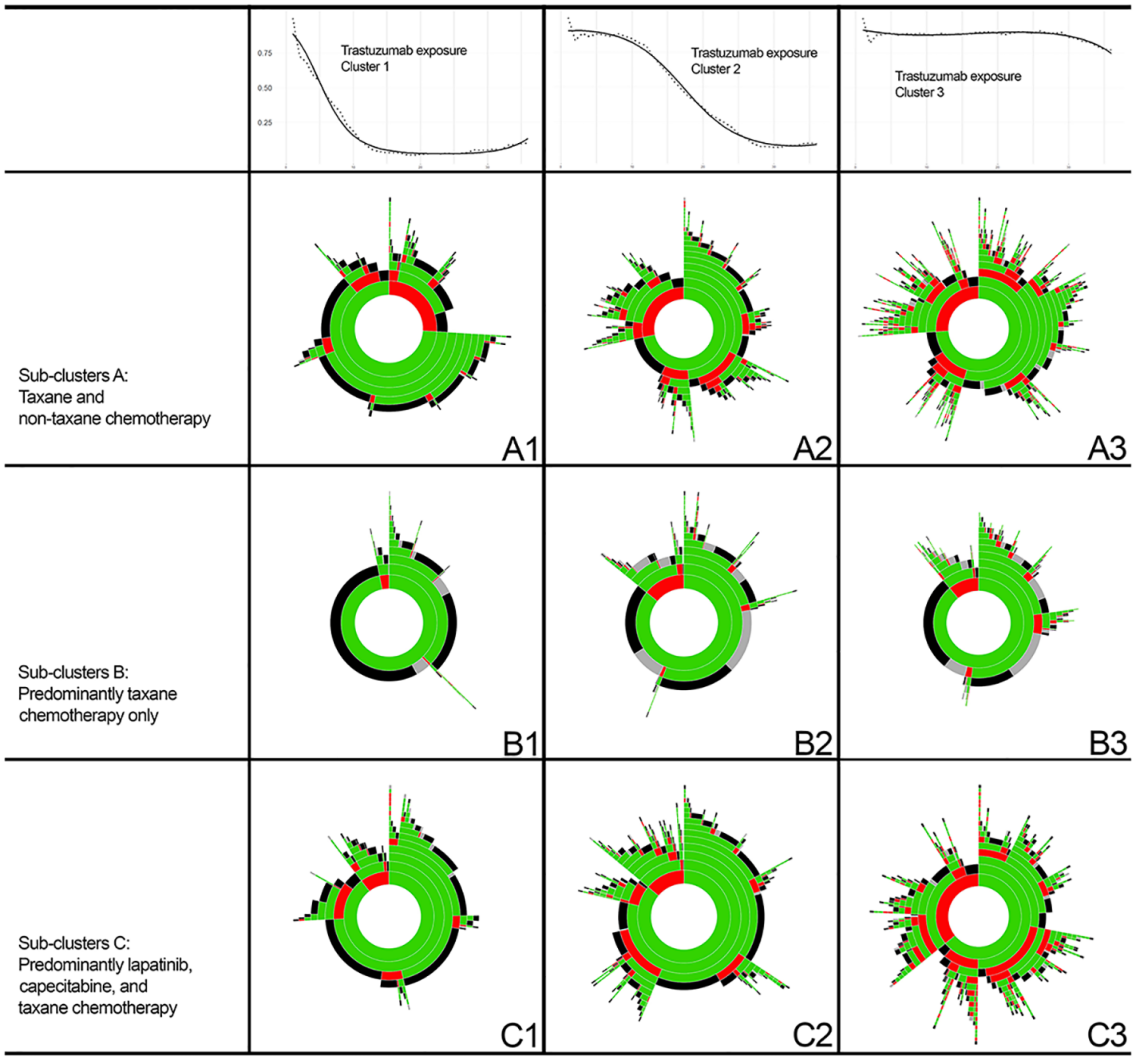
Sequence of treatments by antineoplastic exposure sub-clusters within trastuzumab exposure clusters. Each ring, beginning from the inner ring and moving outwards, represents a new administered treatment and the size of segments within each ring indicates the proportion of patients with that treatment (a larger segment indicates a larger proportion of patients). Green = adherent treatment; red = non-adherent treatment; grey = censor; black = death.

The sunburst graphs in Fig 3 highlight the number and sequence of different treatments dispensed to patients in each sub-cluster. The largest number of treatments were dispensed to patients in sub-clusters A2 –A3 and C2 –C3, with a median number of treatments of three in each sub-cluster. Most non-adherent treatment (red) was observed in a period after treatment initiation, however, in each sub-cluster there were some patients who received non-adherent treatment at the time of treatment initiation (ranging from 11%– 36% of patients in Sub-clusters A1 –A3 and C1 –C3; Table 2). The median proportion of observed survival time from trastuzumab initiation spent on non-adherent treatment, for patients administered non-adherent treatment, ranged from 7%–33% and was highest in Sub-clusters A1 –A3 (Table 2).

## Discussion

Our study highlights the considerable heterogeneity in the real-world treatment of HER2+ MBC and demonstrates that just over one third of Australian patients receiving trastuzumab between 2001 and 2016 received treatment that did not adhere to prescribing restrictions. In Australia, the medicine funding bodies put in place prescribing restrictions to promote cost-effective treatment. This is a delicate balancing act between providing access to new, costly cancer treatments and ensuring the sustainability of public funding. The case of HER2-targeted therapy is particularly interesting because of how effective and well-tolerated these medicines are, and their safety and efficacy when used in combination with a variety of chemotherapeutic partners. When trastuzumab for HER+MBC was approved for public subsidy in Australia the approval was based on its efficacy as demonstrated in pivotal trial where it was used in combination with a taxane, and the restrictions were put in place to align with that trial evidence.

The evidence base around effective treatments is ever evolving, as are advances in targeted therapy more broadly,[21] and treatment guidelines and prescribing restrictions often lag behind this rapidly changing landscape. While a large proportion of our cohort received non-adherent combination treatment, the majority of this non-adherent treatment was generally consistent with emerging clinical trial evidence. Several of the most common combinations we observed—trastuzumab and vinorelbine,[22] trastuzumab and capecitabine,[8] and TCH [23]—all have been found to be effective for HER2+ MBC. It is noteworthy that there was a change to the prescribing restrictions in 2015 permitting trastuzumab use with any concomitant chemotherapy except nab paclitaxel and eribulin in Australia.[6] Trastuzumab plus anthracycline was another common non-adherent combination treatment observed in our cohort, despite evidence of increased risks of cardiotoxic events associated with the use of this combination.[24] And while there is evidence to support the use of trastuzumab and lapatinib combination treatment,[25, 26] restrictions still prohibit its use.

As our definition of adherence depended largely on trastuzumab exposure, we found that patients with longer survival and more trastuzumab exposure had higher proportions of non-adherent treatment and spent a larger proportion of their observed survival time on non-adherent treatment. Thus, the greatest opportunity to observe non-adherent treatment was in trastuzumab exposure Cluster 3, which had the highest median OS estimates, highest median estimates for time on trastuzumab, and the highest rates of non-adherent treatment across each sub-cluster. Conversely, the lowest rates of non-adherent treatment were observed in Cluster 1, which also had the shortest median OS and time on trastuzumab estimates. Similarly, non-adherent lapatinib use necessitated that a patient survived long enough and was healthy enough to receive the medicine, but be ill enough to warrant lapatinib treatment. Most non-adherent lapatinib treatment was lapatinib monotherapy and it was most prevalent in trastuzumab exposure Clusters 1 and 2. These were patients who discontinued trastuzumab or died within 24 months of initiation, and lapatinib use in these patients would seem to align with its indication as second- or late-line treatment. We were unable to assess robustly trastuzumab use beyond progression, but the estimates of time on trastuzumab and later-line non-adherent trastuzumab combination treatment suggest this practice was occurring commonly during the study period.

The use of GBTM and GBMTM to cluster patients—based first on their patterns of trastuzumab treatment, then on their patterns of additional antineoplastic treatment—provided an effective means of organizing the complexity of real-world treatment and allowed an exploration of the HER2+MBC treatment pathway as it related to prescribing restrictions. The procedure highlighted that, while a sizeable number of treatment permutations and combinations are possible with seven different cancer medicines, the observed treatment with these agents (which accounted for 95% cancer medicines dispensed to our cohort) was largely homogenous. Most patients initiated trastuzumab as monotherapy or with a taxane. Patients who received further treatment typically received additional chemotherapy (most often vinorelbine, capecitabine, and/or gemcitabine) or lapatinib (with or without capecitabine). The procedure also produced some unexpected results, such as differences in taxane use within the sub-clusters. Some patients in Sub-clusters A1 –A3 persisted in using taxanes far longer than patients in the other sub-clusters; and the use of first-line platinum agents (a non-adherent combination treatment) was most common in Sub-clusters B1 –B3, particularly within trastuzumab exposure Clusters 2 and 3 (Fig 2). Because GBTM identifies latent clusters based on patterns of treatment as opposed to patient outcomes, there was a wide range of survival outcomes observed in Sub-clusters B1 –B3. Patients in these sub-clusters likely included those who died before, or were too sick to receive additional treatment; but also those patients whose disease responded to trastuzumab and did not require additional antineoplastic treatments. The wide range of survival outcomes is highlighted by the interquartile range estimates for patients in Sub-clusters B1 –B3 (Table 2).

### Strengths & limitations

The data used in this study were collected to provide reimbursement and they lack clinical measures such as performance status, site and extent of metastases, dates of diagnosis and progression, comorbid disease, and adverse events. We are unable to describe how treatment pathways might vary based on these important patient factors. Due to confounding by indication we are unable to examine comparative efficacy of treatments and our OS estimates for the patient sub-clusters should be considered with this limitation in mind. Treatment combinations were based on dispensing records and the assumption that dispensed medicines were administered to patients. Most patients treated for HER2+MBC are treated as outpatients which means their dispensing records for chemotherapies and lapatinib are captured by the PBS; however, some chemotherapy and lapatinib treatments may be administered to hospital inpatients and these treatments would not appear in our PBS data.[27] Therefore, our estimates of non-adherent, trastuzumab combination treatment may under estimate the true prevalence. The strengths of this study include the size of the cohort and representativeness of the national sample, as well as 14.5 years of observation time. Our cohort was selected from all women treated with publicly-funded trastuzumab for HER2+MBC in Australia, which, given the high cost of trastuzumab, likely represents all Australian women treated during the study period.

## Conclusions

The treatment paradigm for HER2+ breast cancer is rapidly evolving, with recent trials exploring the efficacy of multiple HER2-targeted therapy as well as treatment without chemotherapy.[28] Funding these high cost medicines in this dynamic environment is a considerable challenge for payers and Australian prescribing restrictions are in place to promote the cost-effective use of these treatments. Our study highlights that, even during the relatively simpler treatment era of our study period, a substantial amount of treatment did not adhere to prescribing restrictions. As more trials are conducted exploring pertuzumab and T-DM1 in combination with different chemotherapies and other HER2-targeted therapies, the regulation and funding of HER2-targeted treatment will only become more complex.
